# Nasal carriage of *Staphylococcus aureus* in healthy dairy cows in Algeria: antibiotic resistance, enterotoxin genes and biofilm formation

**DOI:** 10.1186/s12917-024-04103-x

**Published:** 2024-06-07

**Authors:** Yacine Titouche, Madjid Akkou, Yasmina Djaoui, Donia Mechoub, Abdelhak Fatihi, Allelen Campaña-Burguet, Pascal Bouchez, Laurence Bouhier, Karim Houali, Carmen Torres, Yacine Nia, Jacques-Antoine Hennekinne

**Affiliations:** 1 Laboratory of Analytical Biochemistry and Biotechnology (LABAB), University Mouloud Mammeri, Tizi Ouzou, Algeria; 2 Laboratory of Biotechnologies Related to Animal Reproduction, Institute of Veterinary Sciences, University of Saad Dahlab, Blida 1.Blida, Tizi Ouzou, Algeria; 3 Laboratory For Food Safety, University Paris Est, Maisons-Alfort, Paris, France, Anses, F-94700 France; 4 Area of Biochemistry and Molecular Biology, OneHealth-UR Research Group, University of La Rioja, Logroño, Spain

**Keywords:** *S*. *aureus*, Healthy cows, Nasal carriage, Antibiotic susceptibility, Enterotoxin genes

## Abstract

**Background:**

*Staphylococcus aureus* can colonize and infect a variety of animal species. In dairy herds, it is one of the leading causes of mastitis cases. The objective of this study was to characterize the *S*. *aureus* isolates recovered from nasal swabs of 249 healthy cows and 21 breeders of 21 dairy farms located in two provinces of Algeria (Tizi Ouzou and Bouira).

**Methods:**

The detection of enterotoxin genes was investigated by multiplex PCRs. Resistance of recovered isolates to 8 antimicrobial agents was determined by disc-diffusion method. The slime production and biofilm formation of *S*. *aureus* isolates were assessed using congo-red agar (CRA) and microtiter-plate assay. Molecular characterization of selected isolates was carried out by *spa*-typing and Multi-Locus-Sequence-Typing (MLST).

**Results:**

*S*. *aureus* was detected in 30/249 (12%) and 6/13 (28.6%) of nasal swabs in cows and breeders, respectively, and a total of 72 isolates were recovered from positive samples (59 isolates from cows and 13 from breeders). Twenty-six of these isolates (36.1%) harbored genes encoding for staphylococcal enterotoxins, including 17/59 (28.8%) isolates from cows and 9/13 (69.2%) from breeders. Moreover, 49.1% and 92.3% of isolates from cows and breeders, respectively, showed penicillin resistance. All isolates were considered as methicillin-susceptible (MSSA). Forty-five (76.3%) of the isolates from cows were slime producers and 52 (88.1%) of them had the ability to form biofilm in microtiter plates. Evidence of a possible zoonotic transmission was observed in two farms, since *S*. *aureus* isolates recovered in these farms from cows and breeders belonged to the same clonal lineage (CC15-ST15-t084 or CC30-ST34-t2228).

**Conclusions:**

Although healthy cows in this study did not harbor methicillin-resistant *S*. *aureus* isolates, the nares of healthy cows could be a reservoir of enterotoxigenic and biofilm producing isolates which could have implications in human and animal health.

## Background

*Staphylococcus aureus* is a significant opportunistic pathogen responsible for severe infections in both humans as well as in livestock species that are economically important [1]. In humans, it can cause a wide range of infections, ranging in severity from superficial skin and soft tissue infections to life-threating endocarditis, bacteriemia, toxic-shock syndrome, and necrotizing pneumonia, among others [2]. In animals, it can cause a large array of diseases with substantial economic impacts in livestock animals, including mastitis in dairy cows and ruminants, joint infections in poultry and surgical site infections in equine, among others [3]. Interestingly, as a commensal bacterium, *S*. *aureus* can colonize its hosts without causing any health issues [4]. It has been reported to colonize 30% of the nares of humans and it can also colonize practically all domesticated farm animals, including pigs, cattle, and poultry, as well as companion animals, such as cats, dogs, and horses. Additionally, it has also been found in wild animals [5].

The development of antimicrobial resistance (AMR) in bacteria has begun to pose a challenge both for clinicians and researchers [6]. The emergence of AMR was associated to the use of antimicrobial agents in humans and animals for therapeutic or prophylactic purposes as well as for animal growth promotion, although the latter is now banned in many countries, but still allowed in others [7]. Numerous zoonotic organisms that are frequently resistant to antibiotics and common causes of foodborne illness are highly prevalent in farm animals, including *S*. *aureus* [8]. *S*. *aureus* has the ability to acquire resistance to different types of antibiotics. For example, methicillin-resistance is acquired through the *mecA* gene (or very unfrequently the *mecC* gene), present in a mobile genetic element called staphylococcal cassette chromosome *mec* (SCC*mec*). The *mec* gene encodes a modified penicillin-binding protein, PBP2a (or PBP2’), which has a low affinity for most β-lactam antibiotics [9]. Methicillin-resistant *S*. *aureus* strains (MRSA) are a significant global cause of infections in both hospital and community settings [1]. The hospital-associated-MRSA (HA-MRSA) strains generally exhibit resistance to numerous antimicrobial agents and carry larger SCC*mec* elements (types I, II and III), whereas community-associated-MRSA (CA-MRSA) strains typically harbor smaller SCC*mec* elements (usually types IV and V) and are generally more susceptible to non-beta-lactam agents [9]. However, the expression of virulence factors, such as Panton-Valentine leukocidin (PVL), appears to be more frequent among CA-MRSA strains [10]. The increasing reports of community infections and the emergence of new virulent clones underscore the crucial importance of identifying potential reservoirs for newly emergent strains in humans to better understand the transmission routes of MRSA strains [1].

As reported, livestock serve as reservoirs for MRSA strains (LA-MRSA), which can be transmitted to humans in close contact with colonized animals [3]. Numerous reports document LA-MRSA transmission from animals to humans, especially those living and working in close proximity with farm animals [11, 12], like farmers and their family members, veterinarians and veterinary students who frequently exposed to sick and healthy animals harboring animal-associated staphylococci [13]. There are some factors influencing LA-MRSA spread, including the frequency of MRSA-carrying animals on the farm and the intensity/duration of animal contact [4]. Furthermore, the age of animals is regarded as a risk factor, with younger pigs more likely colonized with MRSA [14]. Beyond direct human-to-human transmission, MRSA can also disseminate through the food production chain via contaminated animal derived foods like milk and meat. Therefore, handling or consumption of foods of animal origin contaminated with MRSA poses another potential transmission route [13, 15].

In Algeria, a pandemic clone CA-MRSA ST80 PVL-positive has been disseminated in both community and healthcare settings [16–18]. Additionally, other MRSA lineages, such as MRSA-ST8 [19], MRSA-ST5 [20] and MRSA-ST80 [21] have also been isolated from food products. However, there is limited data available on the prevalence and pheno-genotypic characteristics of MSSA and MRSA strains of animal origin. This study aimed to determine the prevalence of *S*. *aureus* in the nasal cavity of healthy cows and breeders (farm owners) and to characterize the obtained isolates phenotypically and genotypically.

## Methods

### Ethics approval and consent to participate

This study was approved by an internal ethics committee of the University Mouloud Mammeri of Tizi Ouzou, Algeria (UMMTO/09/01/2019/Eth-Ani-A102), according to the guidelines of the declaration of the Helsinki. All samples collected from animals and humans were collected under the written informed consent of farm owners and every farmer was provided with a document explaining the purpose and method of sample collection.

### Sample collection

**Fig. 1:**
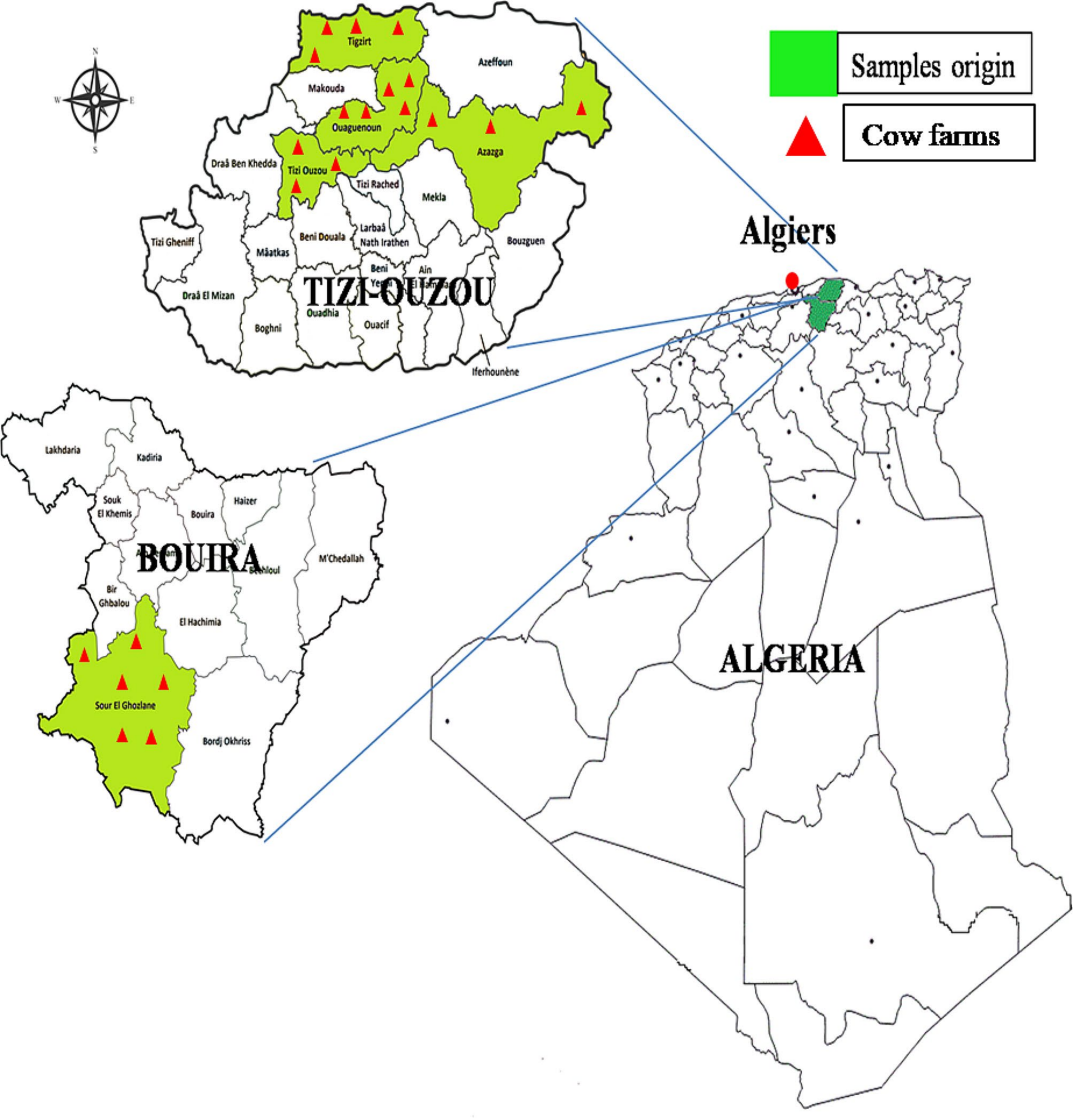
Geographic distribution of the 21 cow farms located at Tizi Ouzou and Bouira provinces of Algeria in which the nasal samples were taken to be analyzed in this study

Between February 2019 and May 2020, a total of 270 nasal samples were obtained, including 249 swabs from cows and 21 from breeders (one sample of breeder from each farm) collected in 21 dairy farms located at different regions of two provinces of Algeria: Tizi Ouzou (Tigzirt, Ouaguenoun, Azazga, and Tizi Ouzou) and Bouira (Sor El Ghozlane) (Fig. 1). The choice of these two provinces was based on the concentration of dairy farms. The farms included in this study were small family farms (maximum of three to fifteen cows per farm) and included Holstein and Montbeliarde cows. The age of the cows ranged from 3 to 10 years with an average of 7 years. The farms were managed by family farmers who breed and take care of their animals. In each farm, we have selected randomly the cows based only on their health state and their treatment with antibiotics. All cows and breeders were considered healthy since at the time of sampling, none presented clinical symptoms of infection and none was treated with antibiotics. Nasal swabs were collected from cows and farm owners on cow farms in which it was obtained the consent for participation. The nasal samples were taken by swabbing both nares of each cow or breeder (one sample per cow or breeder) with a sterile cotton swab (after proper cleaning and disinfection of the external nares with cotton soaked with 70% ethyl alcohol), which was introduced into each nostril (distance of 5 to 10 cm in cows and 2 to 3 cm in breeders to reach the ventral meatus) to collect the nasal mucus. After sampling, the nasal swab was stored in Mueller Hinton broth supplemented with 6.5% NaCl. Samples were placed in cooled containers and immediately transported to the laboratory. All samples were analyzed within 24 h of arrival.

### Microbiological identification of *S*. *aureus* isolates

The swab samples were enriched in 5 ml of Mueller-Hinton broth (Conda Pronadisa, Spain) containing 6.5% NaCl (v/v) and incubated overnight at 37 °C. After incubation, an aliquot of the broth (0.1 ml) was spread on Baird Parker agar (Conda Pronadisa, Spain) supplemented with 5% egg yolk and tellurite (Conda Pronadisa, Spain), and plates were incubated for 24 h to 48 h at 37 °C. Suspected *S*. *aureus* colonies were selected based on their morphology and color (one to five *S*. *aureus* colonies were selected from each positive sample), and subcultured onto brain heart infusion agar (BHIA) (Biokar, France). The obtained isolates were identified as *S*. *aureus* using conventional methods including Gram stain reaction, catalase, coagulase (Biokar, France) and DNase tests. The reference strain *S*. *aureus* ATCC 25,923 was used as positive control to validate the results of all microbiological tests. Phenotypically identified *S*. *aureus* isolates were stored in brain heart infusion broth (BHIB) (Conda Pronadisa, Spain) with 30% glycerol (v/v) at -20 °C for further characterization [19].

### Molecular characterization of *S*. *aureus* isolates

#### DNA extraction

The overnight culture of *S*. *aureus* isolates were submitted to DNA extraction using the InstaGene Kit (Bio Rad, France), according to the manufacturer’s recommendations. DNA concentrations were quantified using a Nanodrop 1000 spectrophotometer (Thermo Scientific, Wilmington, DE) [20].

#### Identification of *S. aureus* isolates by PCR amplification of 23 S rRNA gene

The identification of *S*. *aureus* isolates was carried out using the protocol described by Straub et al. [22]. The resulting PCR products were separated by gel electrophoresis on a 2% (w/v) agarose gel and visualized by staining with ethidium bromide (1 µg ml^− 1^) using the Gel Doc EQ apparatus (Bio-Rad, France). A 1-kb DNA ladder (Promega, Lyon, France) was served as a molecular weight standard. The reference strain *S*. *aureus* FRI 361 was used as positive control [20].

#### Detection of enterotoxin genes by multiplex-PCR

All *S*. *aureus* isolates were tested for the presence of 11 staphylococcal enterotoxin genes using the method described by Roussel et al. [23] and validated by the European reference laboratory for coagulase positive staphylococci (EURL CPS). This involved performing two multiplex PCR reactions. The first PCR detected six “classical” enterotoxin genes (*sea*, *seb*, *sec*, *sed*, *see* and *ser*), and the second PCR detected five newly identified enterotoxin genes (*seg*, *seh*, *sei*, *sej* and *sep*). After PCR amplification, the PCR products were separated by agarose gel electrophoresis (2% agarose) and the bands were visualized using the Gel Doc EQ apparatus (Bio-Rad). Five reference *S*. *aureus* strains (i.e. FRIS6, 374 F, FRI137, FRI326 and FRI361) served as positive controls, as described previously [20].

#### Molecular typing of selected isolates

Selected *S*. *aureus* isolates which shared the same pheno-genotypic characteristics (antimicrobial resistance and enterotoxin gene profiles) and were recovered in both cows and breeders were typed by sequencing the repeat region of the *Staphylococcus* protein A gene (*spa*) obtained by PCR [24]. The obtained sequences were analyzed by Ridom Staph-Type software, by detection and assignment of *spa* repeats (http://spaserver.ridom.de/). Multilocus sequence typing (MLST) was also performed in these selected *S*. *aureus* isolates. The seven house-keeping genes (*arcC*, *aroE*, *glpF*, *gmk*, *pta*, *tpi* and *yqiL*) of the *S*. *aureus* isolates were amplified as previously described [25], and sequence type (ST) was assigned from sequence analyses on the MLST database (http://pubmlst.org/). The clonal complex (CC) of the isolates was assigned according to their *spa* types (when it was possible), or to their sequence types (ST).

### Antimicrobial susceptibility testing

Antimicrobial susceptibility testing was carried out using the disk diffusion method as recommended by the Clinical and Laboratory Standards Institute, CLSI [26]. Eight antimicrobial agents were tested (µg/disk): penicillin G (10 UI), cefoxitin (30), gentamicin (10), tetracycline (30), erythromycin (15), ofloxacin (15), chloramphenicol (30) and trimethoprim/sulfamethoxazole (1.25/23.75). The reference strain *S*. *aureus* ATCC 25,923 was used as control strain in susceptibility testing. After incubation, the inhibition diameters were measured and the strains were categorized as susceptible, intermediate or resistant according to breakpoints of the CLSI [26].

### Detection of biofilm production

#### Congo red agar method (CRA)

To assess the slime production of *S*. *aureus* isolates, the Congo Red Agar method (CRA) described by Freeman et al. [27] was used. In this protocol, a single colony from each *S*. *aureus* isolate was streaked on CRA plates and incubated aerobically at 37 °C for 24 to 48 h. After incubation, isolates that formed black colonies were identified as slime producers, while the non-biofilm producer strains developed red colonies on the CRA plates.

#### Microtiter plate assay (MPA)

The biofilm-forming capacity of *S*. *aureus* isolates was evaluated using a quantitative microtiter plate assay (MPA) as described by Stepanović et al. [28], with some modifications. Initially, *S*. *aureus* isolates were grown in BHIA (Conda Pronadisa, Spain) at 37 °C for 24 h under aerobic conditions. The next day, two colonies were inoculated into 5 ml of trypticase Soy Broth (TSB) (Conda Pronadisa, Spain) supplemented with 1% of Glucose (Sigma-Aldrich, Isère, France) and incubated overnight at 37 °C without shaking. After incubation, the cultures were diluted 1:50 in TSB-1% glucose, and 200 µl of each diluted culture were transferred into three wells of a 96-well, flat-bottomed, tissue culture-treated plate (ProLab Scientific Co Ltd, Zhejiang, China). The reference strain *S*. *aureus* ATCC 25923 was used as positive control and the medium TSB-1% glucose served as a negative control. After overnight incubation at 37 °C, the non-adhered cells were removed from the wells by gently overturning the plate onto paper towels, and the wells were gently washed with phosphate-buffered saline (PBS) three times and allowed to dry. The adherent bacterial cells were fixed with 150 µL of methanol (Honeywell, Seelze, Germany) for 15 min, and the biofilm formed was stained with 0.5% crystal violet (Biochem Chemopharma, Nièvre, France) for 15 min. After washing, the dye bound to the cells was eluted with 95% ethanol, and the biofilm formation was measured at 560 nm using microtiter-plate reader (Gentaur, Paris, France). The biofilm formation was expressed as optical density (OD) values. Each *S*. *aureus* isolate was studied in triplicate in a single experiment. The average OD value of all tested strains (ODs) and negative controls was calculated. Cut-off OD (ODc) is defined as three standard deviations above the mean OD of the negative control. The isolates were classified into four following categories based on the optical density: non-biofilm producer (OD test < ODc), weak biofilm producer (ODc < OD < 2X ODc), moderate biofilm producer (2X ODc < OD < 4X ODc), and strong biofilm producer (4X ODc < OD).

## Results

A total of 249 healthy cows and 21 breeders from 21 dairy herds located in two provinces of Algeria (Tizi Ouzou and Bouira) were screened for *S*. *aureus* nasal carriage. Overall, 12% of the screened cows and 28.6% of the breeders were colonized with *S*. *aureus* (Table 1). A total of 59 and 13 isolates were recovered from cows and breeders (one or two isolates per positive sample), respectively. In all, 26 of these isolates (36.1%) harbored one or more genes encoding for staphylococcal enterotoxins, including 17 (28.8%) isolates from cows and 9 (69.2%) isolates from breeders. In total, ten and four enterotoxin gene profiles were observed in *S*. *aureus* isolates from cows and breeders, respectively. The most detected gene was *seb* (8.5%) in cow isolates. However, *seg* and *sei* genes were the most commonly found in breeder isolates. Other enterotoxin gene profiles were identified with lower frequencies (Table 2). None of the isolates contained the *see* and *sep* genes.

**Table 1:** Number and distribution of cows and breeders nasal samples carrying *S. aureus* isolates in different regions of two provinces of Algeria (Tizi Ouzou and Bouira)

Provinces	Regions	Number of collected samples		Number (%) of samples carrying *S. aureus*	
		Cows	Breeders	Cows	Breeders
Tizi Ouzou	Tigzirt	95	4	11 (11.6)	2 (50)
	Ouaguenoun	60	5	0 (0)	2 (40)
	Azazga	37	3	4 (10.8)	1 (33.3)
	Tizi Ouzou	21	3	4 (19)	1 (33.3)
Bouira	Sor El Ghozlane	36	6	11 (30.5)	0 (0)
Total		249	21	30 (12)	6 (28.6)

**Table 2:** Distribution of enterotoxin genes among the 72 *S*. *aureus* isolates recovered from cow and breeder samples

Enterotoxin genes	Number (%) of *S. aureus* isolates carrying the genes	
	Cows (*n* = 59)	Breeders (*n* = 13)
*seb*	5 (8.5)	0 (0)
*sec*	2 (3.4)	0 (0)
*ser*	1 (1.7)	0 (0)
*sea* + *seb*	1 (1.7)	0 (0)
*seg* + *sei*	2 (3.4)	5 (38.5)
*sec* + *seg* + *sei*	1 (1.7)	1 (7.7)
*sea + seg + sei*	0 (0)	2 (15.4)
*seg* + *seh* + *sei*	1 (1.7)	1 (7.7)
*sec* + *seg* + *seh* + *sei*	1 (1.7)	0 (0)
*sea* + *seb* + *ser* + *seg* + *sei*	1 (1.7)	0 (0)
*sed* + *ser* + *seg* + *sei* + *sej*	2 (3.4)	0 (0)
Total	17 (28.8)	9 (69.2)

After antibiotic susceptibility testing, it was found that 44 (61.1%) of the *S*. *aureus* isolates were resistant to at least one antimicrobial agent. However, only three isolates were multidrug resistant (MDR), and exhibited resistance to penicillin, tetracycline and ofloxacin. Among them, one isolate was recovered from a cow and the other two from breeders. The highest resistance rate was found for penicillin in isolates from cows and breeders, with values of 49.1% and 92.3%, respectively, followed by tetracycline (30.5% and 23.1%, respectively). Resistance to erythromycin, gentamicin and ofloxacin was not so commonly observed (≤ 15%). All isolates were susceptible to sulfamethoxazole/trimethoprim and chloramphenicol (Table 3). All *S*. *aureus* isolates were cefoxitin-susceptible and were considered as methicillin-susceptible (MSSA).

**Table 3:** Antibiotic resistance of the 72 *S*. *aureus* isolates from cows (*n* = 59) and breeders (*n* = 13)

Antibiotics	No (%) of *S. aureus*			
	Resistant		Susceptible	
	Cows	Breeders	Cows	Breeders
Penicillin G	29 (49.1)	12 (92.3)	30 (50.8)	1 (7.7)
Cefoxitin	0 (0)	0 (0)	59 (100)	13 (100)
Chloramphenicol	0 (0)	0 (0)	59 (100)	13 (100)
Erythromycin	4 (6.8)	2 (15.4)	48 (81.3)	10 (76.9)
Gentamicin	1 (1.70)	0 (0)	57 (96.6)	13 (100)
Tetracycline	18 (30.5)	3 (23.1)	36 (61)	10 (76.9)
Sulfamethoxazole/trimethoprim	0 (0)	0 (0)	59 (100)	13 (100)
Ofloxacin	1 (1.70)	2 (15.4)	58 (98.3)	11 (84.6)

A possible transmission of *S*. *aureus* between breeders and cows was shown in two farms (number 4 and 18). In farm 4, the same clonal lineage ST15-t084 was found in *S*. *aureus* isolates from one cow and one breeder, and both isolates shared the same antimicrobial resistance phenotype. Similarly, in farm 18 another clonal lineage (ST34-t228) was found in isolates of the cow and the breeder, and the isolates shared also identical enterotoxin gene profile and resistance phenotype (Table 4).

**Table 4:** Pheno-genotypic characteristics of *S*. *aureus* isolates recovered from cows and breeders of two farms

Isolate number	Source	Farm	*Spa*-type	ST	CC	Enterotoxin gene profile	Antimicrobial resistance phenotype
416	Cow	4	t084	15	15		P-TE-OFX
409	Breeder	4	t084	15	15		P-TE-OFX
434	Cow	18	t2228	34	30	*seg* + *seh* + *sei*	P-E
448	Breeder	18	t2228	34	30	*seg* + *seh* + *sei*	P-E

P : penicillin G ; TE : tetracycline ; OFX : ofloxacin ; E : erythromycin ; ST: sequence type; CC: clonal complex

Biofilm formation ability of tested isolates from cows and breeders was phenotypically assessed by the CRA method. In total, 45 (76.3%) and 10 (76.9%) of the *S*. *aureus* isolates from cows and breeders were found to be slime-producers (Table 5). In MPA, it was observed that most of them showed the ability to produce biofilm, including 52 (88.1%) and 11 (84.6%) isolates from cows and breeders respectively. In cows, 23 (38.9%) isolates showed strong biofilm formation, 12 (20.3%) isolates showed moderate biofilm formation, and 17 (28.8%) isolates showed weak biofilm formation. The remaining isolates (11.9%) lacked the capacity to form biofilm (Table 5). In breeders, 4 (30.8%) of the isolates showed strong biofilm formation, 4 (30.8%) isolates showed moderate biofilm formation, and 3 (23.1%) isolates showed weak biofilm formation. The remaining isolates (15.4%) lacked the capacity to form biofilm (Table 5).

**Table 5:** Distribution of slime and biofilm producing *S*. *aureus* isolates recovered from nasal swabs of healthy cows (*n* = 59) and breeders (*n* = 13)

Criteria			Number and % of isolates	
			Cows	Breeders
Slime producing (CRA performance)	Positive		45 (76.3)	10 (76.9)
	Negative		14 (23.7)	3 (23.1)
Biofilm producing (MPA performance)	Positive	Weak formation	17 (28.8)	3 (23.1)
		Moderate formation	12 (20.3)	4 (30.8)
		Strong formation	23 (38.9)	4 (30.8)
		Total	52 (88.1)	11 (84.6)
	Negative		7 (11.9)	2 (15.4)

CRA : Congo red agar ; MPA : Microtiter-plate assay

## Discussion

Studies on the transmission dynamic of livestock-associated MRSA between different host species have been limited, despite it being a major global concern [29]. However, reports of MRSA infection and colonization have been reported in a range of animal species, including farm and domestic animals [30]. This study aimed to investigate the phenotypic and genotypic characteristics of *S*. *aureus* isolates recovered from nasal swabs of healthy cows and breeders in two provinces of Algeria.

A low rate of nasal carriage of *S*. *aureus* in healthy cows was detected in our study (12%). This result corroborates the findings of previous studies conducted in Algeria [31], Egypt [32], Portugal [33], Morocco [34], Turkey [35], Tunisia [36] and Iran [37], with values of 15%, 4.3%, 13.1%, 9.9%, 3.2%, 1.3% and 5.1%, respectively. In Algeria, Bounar-Kechih et al. [38] also reported a high prevalence rate of *S*. *aureus* in bovine (55%). In the present study, 28.6% of breeders were found colonized by *S*. *aureus*, which agree with the results of Kalayu et al. [39] in Ethiopia and Silva et al. [33] in Portugal. However, a higher rate of *S*. *aureus* was reported by Mourabit et al. [34] in Morocco, with a value of 60%. Various factors might explain the difference in the isolation frequency of *S*. *aureus* through the conducted studies, including livestock density, isolation methods, husbandry practices and geographical conditions [30].

Some isolates from cows (28.8%) in this study carried the genes encoding for staphylococcal enterotoxins, that is in agreement with the results obtained by other authors who have shown the presence of enterotoxin genes in *S*. *aureus* isolates of animal origin [40–42]. However, the prevalence of staphylococcal enterotoxin genes was higher in isolates obtained from breeders than in those isolated from cows. Similarly, El-Ashker et al. [42] have reported a toxinogenic *S*. *aureus* harboring the *luk*-*S/F*-PV, *tst* and the staphylococcal enterotoxin genes among the *S*. *aureus* isolates of human origin. The existence of enterotoxin genes in isolates from healthy cows and breeders may pose a public health hazard to consumers, since these staphylococci can contaminate raw milk during improper milking practices and potentially lead to foodborne illnesses.

In this study, the *S*. *aureus* isolates from healthy cows and breeders were resistant to tetracycline and penicillin with higher rates in relation to other antibiotics, which may be a consequence of the wide use of these antimicrobial agents not only in human medicine but also in veterinary medicine [43]. Our results corroborate those of many authors, who found a high frequency of resistance of *S*. *aureus* isolates to penicillin and/or tetracycline [31, 32, 38, 41, 44]. The use of antibiotics across various domains, including human and veterinary healthcare, agriculture, and other areas, can exert selective pressure that favors the emergence and dissemination of antibiotic-resistant microorganisms [45]. Only small numbers of isolates were resistant to erythromycin, gentamicin and ofloxacin, both for isolates of animal and human origin. The values obtained do not agree with those of Bounar-Kechih et al. [38], who found high resistance rates to enrofloxacin and erythromycin. All isolates were susceptible to chloramphenicol and sulfamethoxazole/trimethoprim. None of the *S. aureus* isolates of this study were MRSA. Our results agree with those of Khemiri et al. [41] and Mourabit et al. [34]. However, many authors have reported the presence of MRSA among *S*. *aureus* isolates from nasal swabs in cows [35, 38, 44, 46].

Healthy cattle can harbor bacterial strains potentially transmissible to humans, especially those in close contact with farm animals such as farm workers, veterinarians, and abattoir workers [43]. *S*. *aureus* may spread from cattle to farm workers through direct contact or indirect exposure within the farm environment [12]. In our study evidence of possible zoonotic transmission of *S*. *aureus* between cows and breeders was observed in two farms. In farm 4, one cow was colonized by *S*. *aureus* ST15/t084 (CC15) and one breeder was also colonized by the same clone. In addition, in farm 18, one cow was colonized by a MSSA of clonal lineage ST34/t2228 (CC30) carrying the *seg*, *seh*, *sei* genes and one breeder was colonized by the same clone harboring the same enterotoxin genes. As known, the lineage ST15, mainly associated to human MSSA isolates, is highly prevalent in African countries, according to the findings of healthcare institutions [47, 48]. However, this lineage has also been reported in cattle [31, 49] and wildlife animals [50]. In a study conducted by Aanensen et al. [51], six major CC were observed among MSSA lineages in Europe, including CC5, CC22, CC8, CC30, CC45 and CC15, with CC5 the most observed. However, Tavares et al. [52] reported that the CC30 was the most common clonal complex found in Portuguese MSSA isolates.

Biofilm formation is considered as a way for microorganisms to adapt to their environment, involving physiological changes from that observed in the planktonic mode of growth [53]. It represents an important virulence factor, enabling microbial survival in food production facilities and persistent colonization of biomaterials used in implanted medical devices [54]. This also applies to *S*. *aureus*, which has the ability to produce biofilms, as part of its normal life cycle [55]. As known, the persistence of staphylococci in food processing environments is linked to their ability to form biofilms on abiotic surfaces [56]. However, little published works were available concerning the biofilm formation ability of *S*. *aureus* isolates of animal origin, and the majority of existing studies in this area have focused specifically on *S*. *aureus* strains associated with bovine mastitis [57]. In this study we have used two techniques to evaluate the capacity of recovered *S. aureus* isolates to produce biofilms in vitro; among these isolates, 45 (76.3%) from cows and 10 (76.9%) from breeders were biofilm producers by CRA plate method. Our results agree with those of Achek et al. [58] and Ballah et al. [59], who reported that 70.9% and 89%, respectively, of *S*. *aureus* isolates derived from different origins, including animals, food and humans, were biofilm producers. A similar result was obtained by Vasileiou et al. [60] in *S*. *aureus* isolates from subclinical mastitis in sheep, with value of 69.1%. Although the CRA test is not considered the most sensitive for assessing biofilm formation, this simple qualitative phenotypic test was used for its acceptable levels of sensitivity and specificity [58, 61]. Nonetheless, it is important to note that the production of slime by *Staphylococcus* species can be influenced by various factors, including the presence of glucose and sodium chloride [62]. The MPA test revealed that most of the isolates (88.1%) showed the ability to produce biofilms. Silva et al. [57], found that all *S*. *aureus* isolates recovered from different animal species, including pets, livestock and wild animals, produced biofilm; however, isolates from pigs and cows were the least biofilm producers among all tested animals, which does not agree with our findings. Studies conducted with *S*. *aureus* isolated from bovine mastitis showed that most isolates were biofilm producers [63, 64]. In food industry, biofilm formation is highly undesirable for sanitary and safety reasons due to the possible adherence of food spoilage or pathogenic microorganisms to food or food surfaces, making their removal more difficult and raising the possibility of cross contamination [65]. Additionally, biofilms offer an environment with a high cell density and a greater innate ability for the exchange of mobile genetic elements, which are involved in the transfer of antibiotic resistance [66]. For this, biofilm formation by *Staphylococcus* spp. confers an evolutionary advantage to these microorganisms, as it enhances their resistance to harsh environmental conditions, such as exposure to antimicrobial agents, sanitizers, and desiccation [67].

There are various limitations to our study. Firstly, the nasal carriage of *S*. *aureus* in cows and breeders could be transient, making it difficult to detect at the time of sampling. Another limitation on our study is the small sample size, which likely had an impact on the quality of results. Further extensive research encompassing a greater number of cow farms collected at different geographical locations might provide additional insights into the nasal carriage of *S*. *aureus* in dairy cows. Furthermore, we have screened only staphylococcal enterotoxin genes in all recovered *S*. *aureus* isolates. However, the isolated *S*. *aureus* strains could harbor additional virulence factors which are implicated in broad range of clinical infections in both animals and humans.

## Conclusion

The results of this study demonstrated a low prevalence of *S*. *aureus* in the nares of healthy cows, and the absence of MRSA among them. However, most isolated strains were biofilm producers and some of them harbored enterotoxin genes that could have public health implications. Our findings highlight the importance of surveillance studies among healthy animals to gain knowledge in the genetic lineages of *S*. *aureus* that may constitute a zoonotic risk for a better understanding of *S*. *aureus* transmission routes and for implementing adequate control measures.

## Data Availability

The datasets used and/or analysed during the current study are available from the corresponding author on reasonable request.
